# Analysis of Sheep Wool-Based Composites for Building Insulation

**DOI:** 10.3390/polym14102109

**Published:** 2022-05-22

**Authors:** Tünde-Orsolya Dénes, Raluca Iştoan, Daniela Roxana Tǎmaş-Gavrea, Daniela Lucia Manea, Andreea Hegyi, Florin Popa, Ovidiu Vasile

**Affiliations:** 1 Department of Civil Engineering and Management, Technical University of Cluj-Napoca, 400020 Cluj-Napoca, Romania; roxana.tamas@ccm.utcluj.ro (D.R.T.-G.); daniela.manea@ccm.utcluj.ro (D.L.M.); 2 NIRD URBAN-INCERC Cluj-Napoca Branch, 400524 Cluj-Napoca, Romania; andreea.hegyi@incerc-cluj.ro; 3 Department of Materials Science and Engineering, Technical University of Cluj-Napoca, 400641 Cluj-Napoca, Romania; florin.popa@stm.utcluj.ro; 4 Department of Mechanics, University Politehnica of Bucharest, 060042 Bucharest, Romania; ovidiu_vasile2002@yahoo.co.uk

**Keywords:** natural fibre composite, thermal conductivity, latex, resin, building insulation, sustainability

## Abstract

The aim of this paper is to propose ecological thermal insulation materials that meet the goals of sustainability but also fulfill the imposed thermal performance requirements. This paper studies new composite materials based on sheep wool from the perspective of thermal conductivity. The composites were prepared using two types of binder: acrylic-polyurethane resin and natural rubber latex, which were applied to the wool fibres through different methods and percentages. Based on the obtained results of thermal conductivity, two types of samples were selected for further analysis, which aimed to determine the microstructure, chemical composition, water absorption, attack of microorganisms, water vapour permeability, hygrothermal adsorption characteristics and sound absorption of the samples. In order to analyse the variation of thermal conductivity, the following parameters were taken into account: thickness, density, type of binder and percentage of binder. Following the obtained results, it was observed that the value of the thermal conductivity of the samples varies between 0.0324 and 0.0436 W/mK. It was found that all the samples prepared and analysed in this study fulfil the national criteria for the thermal performance of thermal insulation material. After conducting the in-depth analysis of the two selected sample types, it was concluded that both materials have good sound absorption characteristics over the considered frequency range. In addition, as it was expected from the natural fibres, the samples had low resistance against the attack of microorganisms and water-related tests.

## 1. Introduction

Climate change and global warming are phenomena that result from the accumulation of CO_2_ in the atmosphere, mainly caused by the process of burning fossil fuels. The concentration of CO_2_ in the atmosphere is constantly increasing due to the increasing energy demand [1]. The construction sector is responsible for 36–40% of total energy consumption, 39% of total CO_2_ emissions, 17–25% of water consumption, 25% of wooden material consumption and produces waste that is between 45 and 65% of total landfilled waste [2,3,4]. Thus, it is understandable the need to propose sustainable construction technologies and materials. These new materials should correspond to the three basic dimensions of sustainability: ecological, by minimising the impact on the natural environment; economic, by using materials and processes with low costs; and social, by ensuring human safety, health and prosperity.

It can be stated that buildings are a major source of energy consumption. However, with adequate thermal insulation of a building, a reduction in fuel consumption of 25–50% [5] and reduction in CO_2_ emissions and energy requirements for heating and cooling of the building [6] can be achieved. Thermal conductivity is an important factor in choosing the type of thermal insulation material, and in the case of these materials, its value is less than 0.065 W/mK, as indicated by the Romanian Norm C107/2 [7]. In addition to low thermal conductivity, a thermal insulation material is characterised by thermal resistance greater than 0.50 m^2^K/W [7].

For a building’s thermal insulation, researchers have developed a wide variety of natural solutions, such as reed or straw bale [8], feather [9] and sheep wool [10] based materials. Sheep wool has good insulating properties due to the micro-cavities formed between the fibres and the tubular structure of the fibres. During the cold season, it contributes to increasing indoor temperatures, and during the hot season, it maintains low temperatures inside the building [11]. Under standard conditions, the thermal conductivity of sheep wool varies between 0.038 and 0.054 W/mK, but the value of thermal conductivity increases with increasing moisture content in the mass of the material or by increasing bulk density [12].

Wool fibres have several unique and interesting properties. Among natural fibres, wool fibres have a unique property of binding, called felting, which occurs when an external directional force is applied over the fibres causing them to become tangled [13]. Wool fibres are hygroscopic, so they absorb moisture from the air. Moisture adsorption by wool fibres is manifested by hysteresis [14]; that is, by changing the external conditions, the adsorbed moisture is released by desorption. Among the common pollutants, wool can absorb and neutralise compounds such as formaldehyde, thus improving indoor air quality [15]. Wool fibres do not contribute to the spreading of flames [11], mainly due to their high nitrogen content, high humidity content and high temperature of ignition (560 °C).

There are various manufacturing processes through which a thermal insulation material from sheep wool can be obtained. The process called wet felting consists of using moisture and friction to bind the fibres together to obtain a denser material [16]. The method of needle felting of a mixture of recycled wool fibres and polyester fibres was described in [17], obtaining a material with low thermal conductivity, between 0.032 and 0.033 W/mK. The product obtained by combining thermal and chemical methods for the partial degradation of fibres in order to obtain a protein-based adhesive that sticks the remaining fibres, was presented in [18]. The use of wool textile waste by preparing a composite thermal insulation material in which the textile fibres are bonded with a chitosan solution was presented by Rubino et al. [19]. The preparation of wool and polypropylene fibre composites, by carding the two types of fibres, followed by hot pressing of the mixture, was presented by Guna et al. [20]. The thermal conductivity of the resulting panels ranged from 0.058 to 0.083 W/mK. The thermal properties of a composite based on clay and wool fibres, respectively, and the possibility of using this material in the field of constructions was studied by [21]. Commercial products usually use a synthetic binder such as polyester [22], which by heating forms a stable matrix [23], the percentage of synthetic material being between 5 and 15% [13].

The purpose of this study is to bring this by-product of the agricultural sector into the field of constructions by creating an insulating material in order to improve the sustainability credentials of buildings. The raw materials used in this study are available worldwide. The produced samples require low energy consumption and a relatively simple infrastructure to prepare them, and thus have a low cost of production, but possess good acoustic and thermal properties.

The unique properties of sheep wool are widely recognised, and several attempts were made to integrate it into the field of building materials as a stand-alone material, reinforcement or part of a composite. In spite of the many possibilities of successful sheep wool utilisation, the preparation methods of wool composites or the used binder types could be further researched and expanded. This study contributes to overcoming this limitation by presenting new preparation methods (spraying and mixing) and binder types (resin and latex) that can be employed in realising novel wool-based composites. The originality of the present study can be synthesised as a novel approach to create natural and ecologically-friendly materials whose properties are assessed in a complex way.

Thus, the aim of this paper is to prepare and analyse composites based on sheep wool fibres from the main perspective of thermal conductivity, intended for use in the field of constructions with a thermal insulating role. The composites were prepared using two types of binder: synthetic (acrylic-polyurethane resin) and natural (natural rubber latex). Thus, the influence of density, thickness, binder type and binder percentage on the thermal conductivity and thermal resistance of the prepared samples was analysed. Based on the values of thermal conductivity and considerations such as visual aspect, ease of fabrication, density and quantity of binder used, two types of composites were selected for further analysis. These investigations were based on determining the microscopical and chemical characteristics, water absorption, attack of microorganisms, water vapour transmission properties, hygroscopic characteristics and acoustic absorption.

The paper is structured in five parts. Section 1 gives a short introduction and overview of wool’s properties and wool-based composites. Section 2 presents the materials and methods used in this study. Section 3 contains all the obtained results of this experiment: Section 3.1 holds the results of thermal conductivity analysis (i.e., the first stage of this experimental program) and Section 3.2, Section 3.3, Section 3.4, Section 3.5, Section 3.6, Section 3.7 and Section 3.8 hold the results of the complementary investigations (i.e., the second stage of this experimental program). Section 4 contains a short discussion on the main results in the view of the relevant literature. Section 5 gives the general conclusions of this study.

## 2. Materials and Methods of Investigation

The following materials were used in this study: sheep wool, acrylic-polyurethane resin, and natural rubber latex. Using these materials, composite panels based on sheep wool and binders were prepared for use with a thermal insulating role in buildings. In this regard, the experimental program was divided into two stages. In the first stage, for the made composites (denoted P1–P24), the thermal conductivity coefficient was determined. Given that the recipes designed were similar, it was decided that in-depth investigations should be carried out on the most representative recipes. Thus, based on the results of thermal conductivity and other factors, such as low density, ease of fabrication, or low binder percentage, recipes P3 and P13 were chosen for further investigations.

### 2.1. Materials

**Figure 1:**
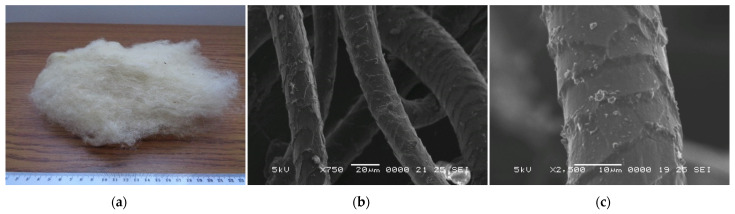
Fibres used for the wool-based composite panels: (**a**) overall aspect of wool fibres; SEM images with a magnification of (**b**) ×750 and (**c**) ×2500.

The commercially available wool (presented in Figure 1) was obtained from the Romanian sheep breed of “Ţigaie”. During processing, the wool was washed and carded. In the composition of the wool-based panels, the wool mattresses were cut to the final size of the samples (i.e., 200 × 200 mm, for the rectangular-shaped samples; 100 mm and 63.5 mm in diameter for the circular samples).

**Figure 2:**
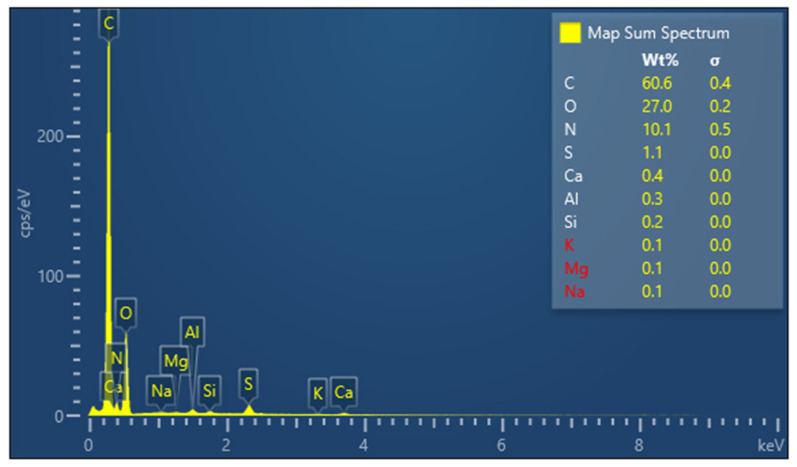
Percentage distribution of the component elements of the wool fibres.

From the SEM images (Figure 1b,c) the outer layer of the fibres, which consists of scale-like cells, can be distinguished, which could contribute to the improvement of adherence between the fibres and binder. Some impurities can also be identified on the surface of the fibres due to the fact that they were not treated with any substance prior to their use in the experimental program. From the EDX analysis (Figure 2), the fibre components can be observed and their distribution in the studied sample. Thus, the elements detected are: carbon in 60.6%, oxygen in 27.0%, nitrogen in 10.1%, sulphur in 1.1%, calcium in 0.4%, aluminium in 0.3%, and silicon in 0.2%. In addition to the elements of wool identified in the literature (carbon, oxygen, nitrogen and sulphur, which make up to 98.8% of the total mass of fibres), other chemical compounds were also identified in small amounts (1.2%) in the analysis of the fibres used in this study.

The resin used is a one-component solution based on acrylic-polyurethane resin and water, commercially available (produced by Köber S.R.L., Săvinești, Romania). According to the manufacturer’s data [24], it does not emit toxic or flammable vapours and does not contain or release formaldehyde after drying. The resin’s characteristics are presented in Table 1.

**Figure 3:**
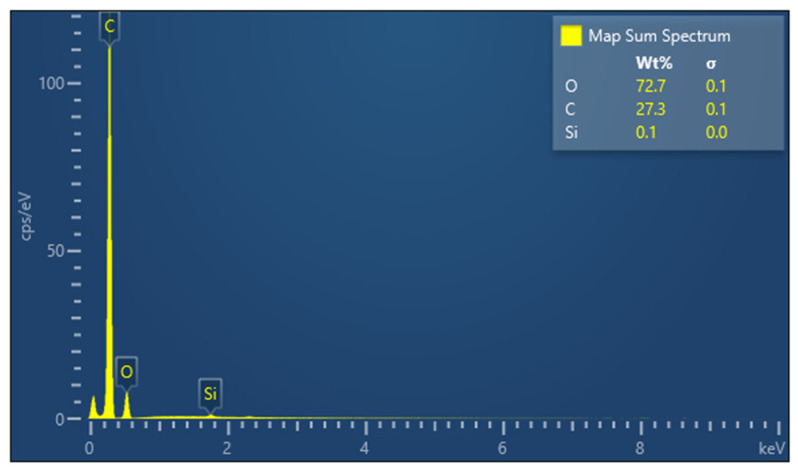
Percentage distribution of the component elements of the resin.

Figure 3 shows the EDX analysis of the resin and, respectively, the percentage distribution of each element in the mass of the analysed sample. The EDX analysis identifies the components of the polyurethane resin as: oxygen, carbon, and silicon.

The natural rubber latex used is a one-component, commercially available solution (produced by Prochima Srl, Colli al Metauro, Italy). According to the manufacturer’s data [25], it is a prevulcanised product, saturated with ammonia and stabilised with antioxidants, being a 61% solid solution. The latex characteristics are presented in Table 1. In order to obtain a suitable workability, the latex was diluted with water in a ratio of latex:water of 1:2.

**Figure 4:**
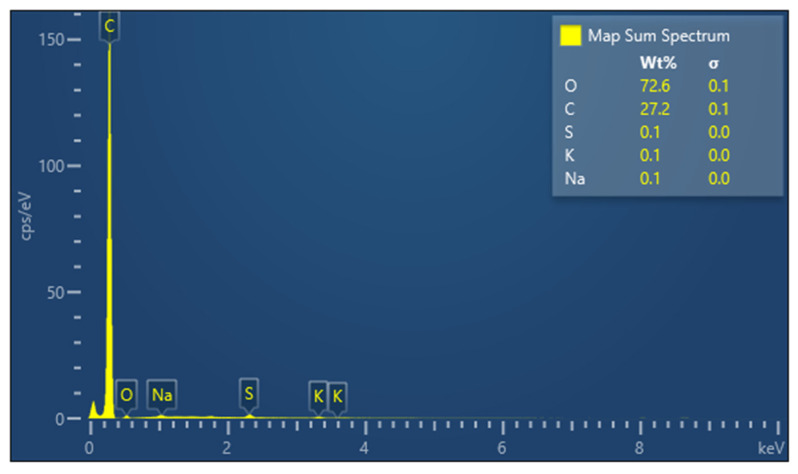
Percentage distribution of the component elements of the diluted latex.

Figure 4 shows the EDX analysis of the latex and the distribution of each element in the sample. The identified components of the latex are as follows: oxygen, carbon, sulphur, potassium, and sodium.

### 2.2. Samples Preparation

In this study, wool fibre-based panels were made with a natural (rubber latex) or synthetic binder (acrylic-polyurethane resin). In order to carry out the experimental program, specimens with the following dimensions were prepared: 200 × 200 mm with a thickness of 30, 40 and 50 mm for thermal conductivity measurement, Φ63.5 mm with a thickness of 30, 40 and 50 mm for the acoustic absorption measurement, 100 × 100 mm with a thickness of 30, 40 and 50 mm for water absorption and hygroscopic properties’ measurement, Φ100 mm with a thickness of 50 mm for the water vapour permeability measurement, respectively. The samples were designed according to the density of wool fibres in the composite material. The procedure for preparing the samples consisted of the following steps: uniformly distributing the wool mattresses on a flat surface; cutting mattresses to the default size; mechanically spraying the binder over the fibres, or mixing the fibres with the binder, depending on the preparation method (described in the following paragraphs); placing the mattresses with the binder in the mould; pressing the mould with a glass sheet. The samples were partially unmoulded after 24 h to facilitate the hardening of the binder, and total unmoulding was performed after 7 days. The recipes for the sample preparation are presented in Table 2. For each determination, three samples were prepared and tested for all of the recipes.

**Figure 5:**
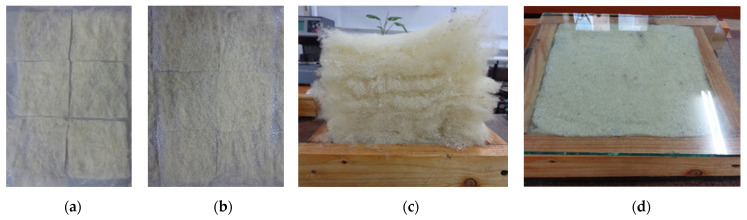
Making the specimens P1–P12 by spraying the binder over the fibres: (**a**) fibre mats cut to size; (**b**) binder sprayed over fibres; (**c**) mats arranged in formwork; (**d**) pressed formwork.

Two general methods of specimen preparation, defined by the binder application technology over the fibres, were used to obtain the samples, regardless of the nature of the binder. Thus, in the first method, the binder was applied by spraying over the fibres. An attempt was made to reduce the thickness of the wool layers in order to achieve a more uniform distribution of the sprayed binder through the specimen mass. Thus, mats with a maximum thickness of 1 cm were prepared. The sprayed samples are shown in Figure 5.

**Figure 6:**
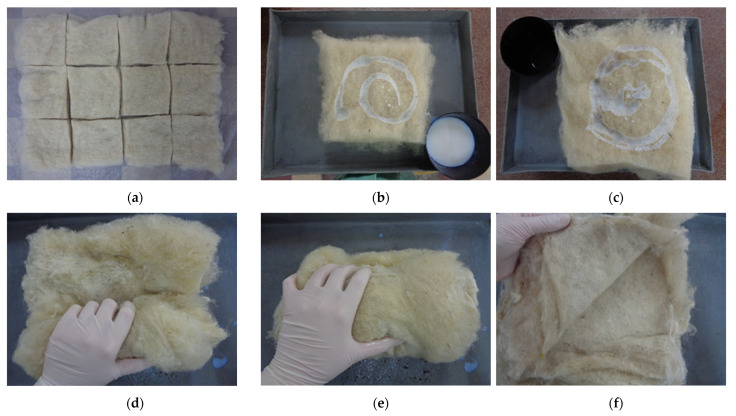
Making the specimens P13–P24 by mixing the binder with fibres: (**a**) fibres cut to size; (**b**,**c**) sprinkling binder over fibres; (**d**,**e**) wrapping the fibres-binder assembly; (**f**) resulting layers.

In the second method of making the wool-binder samples, the panels were obtained by mixing the component materials. The preparation of the samples started by distributing the required amount of wool on a flat surface, after which the mats were cut to the final panel size. The thickness of the mats at this stage was not relevant because the carded fibres of the mats were arranged in one direction and the thickness control of the individual layers was easier after the mixing process. After preparation of the wool mats, they were transferred one by one to a metal tray where they were sprinkled with a small amount of binder, the sprinkled mats being arranged one on top of the other. A cylinder was formed by tightly winding the layers together, starting the process at a random side of the assembly. The cylinder was then unrolled, and the winding of the layers on the opposite side of the assembly was repeated. These steps of wrap–unwrap–wrap were carried out for the 2 opposite sides and 2 diagonals until it was found that the dispersion of the binder was uniform. This obtained assembly was easily unrolled into thin layers, which were arranged in a formwork. The making of the samples by mixing is shown in Figure 6.

### 2.3. Investigation Methods

The determination of thermal conductivity and thermal resistance was carried out according to EN 12,667 [26], using a Lasercomp FOX 200 heat flow meter (TA Instruments, New Castle, DE, USA). For this determination, specimens with dimensions of 200 × 200 mm and thicknesses of 30, 40, 50 mm were prepared. The specimen was dried in an oven, drying being considered complete when the difference between two successive weighings was less than 0.1 g. Thermal properties were evaluated at a temperature difference of 10 °C. The apparatus implicitly determines the following properties: heat flux density, thermal resistance and thermal conductivity.

The microscopic analysis was performed using a scanning electron microscope (SEM), type JSM-5600 LV (Jeol, Tokyo, Japan). Microscopic analysis is coupled with chemical microanalysis, called energy dispersive X-ray spectroscopy (EDX). The determination of the elemental chemical analysis of the samples was carried out using an SEM equipped with an EDX spectrometer, type UltimMAX65, the data being processed in Aztec software (Oxford Instruments, Abingdon, UK). The EDX method, coupled with SEM analysis, provides information related to the chemical composition of the sample and the distribution of elements in a given area. The samples used for the determination were taken from characteristic sections of the specimens, avoiding their outer surfaces and edges. The samples taken were glued by means of a double adhesive carbon tape on metallic supports. Due to the fact that wool fibres do not conduct electrical signals and are sensitive to radiation, it was necessary to metallize the samples. The process consists of depositing a layer of carbon on the surface of the sample by evaporating a carbon filament using a Desk V (Denton Vacuum, Moorestown, NJ, USA)-type cathode sputtering apparatus.

The determination of short-term water absorption by partial immersion was carried out according to EN 12087 [27]. The principle of the method is to determine the mass growth of specimens partially immersed in water for a specified period of time (24 h). After determining the initial mass of the specimen, it was placed in the test assembly. The lower face of the specimens was immersed in water to a depth of 10 mm, the water level being kept constant during the determination. After 24 h the specimen was removed from the test assembly, excess water was removed by draining, and finally, the specimen mass was recorded.

The determination of the action of microorganisms was carried out according to EN ISO 846 [28]. The principle of the method consists of conditioning the specimens in an environment favourable to the growth of microorganisms and visually assessing the intensity of their growth. In this determination, the specimens were maintained in an environment with a relative humidity of 95%. Visual examination of the samples was carried out at 4 and 6 weeks after the start of the test using a stereomicroscope. The following scale from 0 to 5 was used to assess the intensity of growth of the microorganisms: category 0—no visible growth when assessed with the microscope, category 1—no growth visible to the naked eye, but growth is visible when assessed with the microscope; category 2—visible growth with the naked eye, covering maximum 25% of the exposed surface; category 3—visible growth with the naked eye, covering maximum 50% of the exposed surface; category 4—considerable growth, covering more than 50% of the exposed surface; category 5—strong growth, covering the whole exposed surface. After assessing the intensity of growth of microorganisms and placing the material in a category, the results of the determination can be interpreted as follows: for category 0—the material does not favour the growth of microorganisms; for category 1—the material contains limited sources of nutrients for the growth of microorganisms; for categories 2 to 5—the material is not resistant to attack by microorganisms and contains nutrients for their growth.

The determination of water vapour transmission characteristics was carried out according to EN 12,086 [29]. Circular specimens with a diameter of 100 mm and a thickness of 50 mm were prepared for this test. The specimen is sealed on the open face of the test vessel, in which a salt solution was previously placed. The prepared assembly is placed in a room with controllable temperature and relative humidity. The water vapour creates a flow through the specimen mass due to the partial pressure difference of the water vapour between the test assembly and the chamber atmosphere. By successive weighings of the test assembly, the rate of transmission of the water vapour at the steady-state can be determined.

The determination of hygroscopic adsorption characteristics was carried out according to EN ISO 12571 [30]. Both the sorption curve and the desorption curve were recorded in the determination. Specimens with dimensions of 100 × 100 × 30 mm, 100 × 100 × 40 mm and 100 × 100 × 50 mm were prepared. To plot the sorption curve, the specimen is dried until the constant mass is established. The test specimen is successively placed in a series of test media of constant temperature and increasing relative humidity compared with the previous medium. The moisture content of the test specimen is determined when the equilibrium and constant mass have been reached in the medium considered. The same procedure was followed for all chosen test media. The sorption curve was plotted after obtaining the moisture content of the specimen for each relative humidity. The test media had a relative humidity of 30, 50, 80 and 95%, respectively. For the desorption curve, the first conditioning medium has a relative humidity of 95%, which corresponds to the last point on the sorption curve. The test specimen is placed successively in a series of test media of constant temperature and decreasing relative humidity compared to the previous medium. For all test media chosen, the moisture content of the test specimen is determined when equilibrium is reached in the medium considered. After the sample conditioning is complete, the specimen is dried to constant mass. The desorption curve is plotted after obtaining the moisture content of the specimen for each relative humidity.

The determination of sound absorption was carried out according to EN ISO 10534-2 [31], using an impedance tube type 4206A (Brüel&Kjær, Nærum, Denmark), over the frequency range 100–3200 Hz applying the transfer function method. For this determination, specimens of 63.5 mm diameter and 30, 40 and 50 mm thickness were prepared. The sound source is placed at one end of the tube and the test sample is placed at the other end. Sound waves generated by the source propagate along the tube, encounter the surface of the sample and are reflected. The interference field generated is broken down by measuring the sound pressure at two fixed positions using two microphones mounted on the tube wall. The complex sound transfer function is calculated using a signal analyser, with which the sound absorption coefficient at normal incidence can be obtained.

## 3. Results

This section presents the results of the tests performed. It should be noted that thermal conductivity determinations were carried out for all samples (P1–P24), while the rest of the determinations were carried out for samples P3 and P13.

### 3.1. Thermal Conductivity and Thermal Resistance

In the following sections, the effect of sample thickness and density, binder type and percentage on the values of thermal conductivity will be presented and discussed.

#### 3.1.1. Effect of Sample Thickness

**Figure 7:**
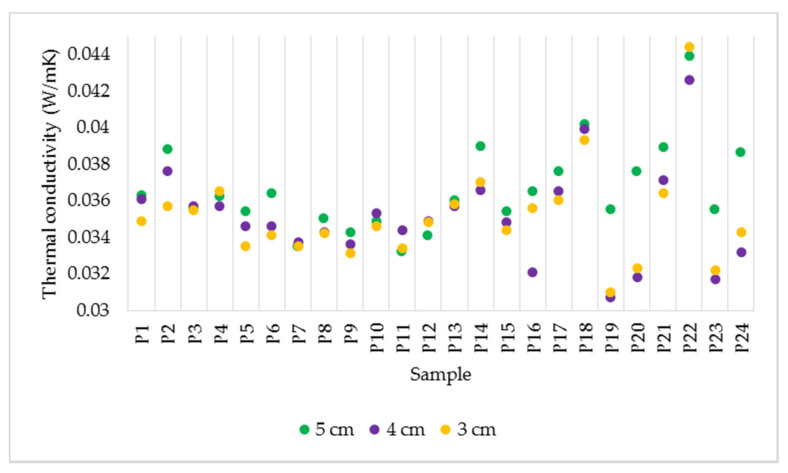
Variation of thermal conductivity for the samples with thicknesses of 5, 4 and 3 cm.

The increase in thickness does not influence the value of thermal conductivity (Figure 7). This aspect comes from the definition of conductivity, according to which this parameter depends on the surface and the temperature gradient perpendicular to the surface. Considering the three thicknesses of materials, however, it was found that higher values were obtained for the 5 cm thick samples in most cases. The different values recorded for the same specimens with different thicknesses can be attributed to the inhomogeneity of the material. As each test piece was made by hand, there is a possibility that the composition of the material is not uniform. From Figure 7, it is observed that for the denser samples made by mixing (P13–P24), the difference in value between thicknesses is more accentuated, while in the case of less dense samples obtained by spraying (P1–P12), the divergence between the measured values in the three cases is less pronounced.

**Figure 8:**
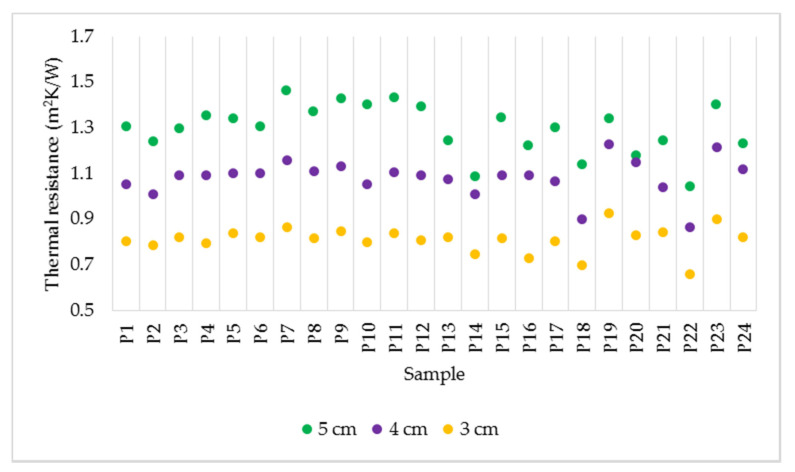
Variation of thermal resistance for the samples with thicknesses of 5, 4 and 3 cm.

Analysing the case of thermal resistance in Figure 8, a significant influence of the thickness of the specimen is noticed. As the thermal resistance is directly proportional to thickness, the maximum values were recorded for the thickest specimens. It was observed that in the case of specimens with a thickness of 3 cm, the variation of the thermal resistance value is almost linear for the samples obtained by spraying.

#### 3.1.2. Effect of Sample Density

**Figure 9:**
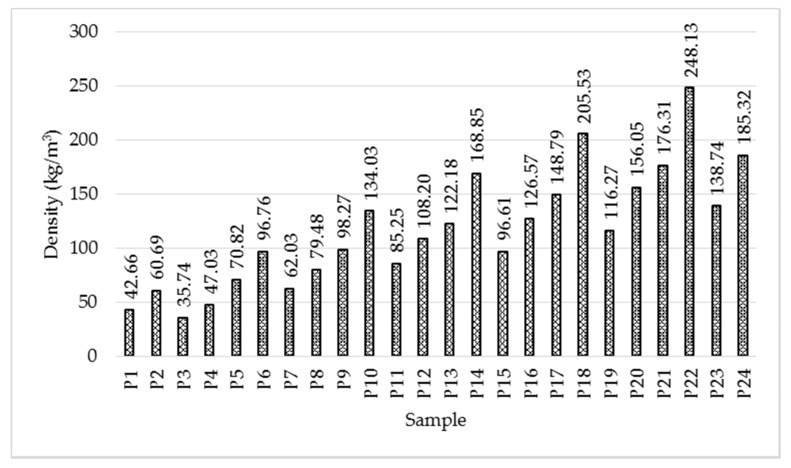
Variation of density of the tested samples; average values.

**Figure 10:**
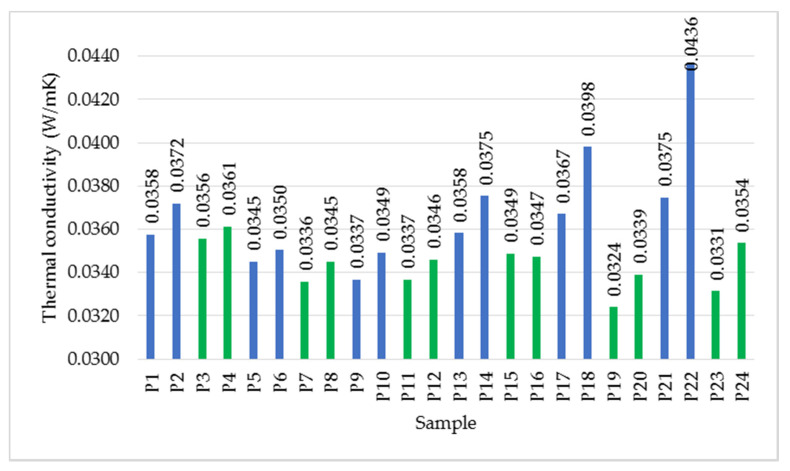
Variation of thermal conductivity, average values. Notation: blue—samples made with resin, green—samples made with latex.

In order to evaluate the effect of density (Figure 9) on the thermal conductivity, the average values for the three thickness categories will be considered. From Figure 10, it is observed that for the samples with lower density made by spraying (P1–P12), the variation between thermal conductivity and density is almost inversely proportional. Instead, analysing the set of samples made by mixing (P13–P24), a significant difference can be found between the samples, the variation being of two types: directly and inversely proportional, depending on the binder used.

The minimum value of thermal conductivity, of 0.0324 W/mK, was obtained for sample P19, with a density of 116.27 kg/m^3^, followed by P23, with 0.0331 W/mK for 138.74 kg/m^3^.

**Figure 11:**
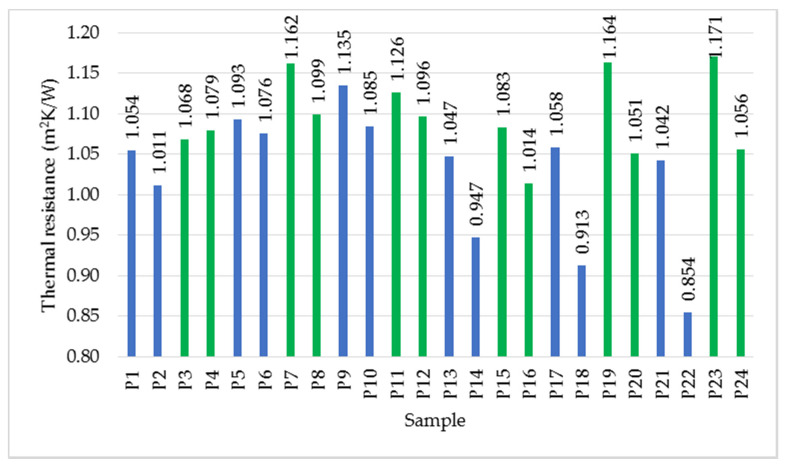
Variation of thermal resistance, average values. Notation: blue—samples made with resin, green—samples made with latex.

Regarding thermal resistance (Figure 11), the highest value, 1.171 m^2^K/W, was measured for P23, followed by P19, with a value of 1.164 m^2^K/W.

#### 3.1.3. Effect of Binder Type

Analysing Figure 10, the significant influence of the binder type on thermal conductivity is observed. In the case of synthetic resin binder, the variation of thermal conductivity as a function of density is nonlinear, the variation curve having a minimum point, for samples P9 and P10 (0.0337 and 0.0349 W/mK). On the other hand, in the case of the natural latex binder, the nonlinear curve presents two minimum points: for P7 and P8 and P19 and P20, the latter set having lower values (0.0324 and 0.0339 W/mK).

It is also observed that, considering the same density category, the samples prepared with a natural binder show a lower thermal conductivity, the difference being much more pronounced at higher density. The thermal resistance varies in a similar way, and the value is higher in the case of samples prepared with a natural binder.

#### 3.1.4. Effect of Binder Percentage

Analysing the effect of binder percentage in the composite, it was found that the higher the percentage of binder, the higher the value of thermal conductivity, regardless of the type of binder used. It was noticed that by increasing the density, the differences between the values of thermal conductivity due to the increase in the binder percentage are more pronounced. Deviations are more significant in the case of samples prepared with synthetic resin.

### 3.2. Microscopic Analysis

**Figure 12:**
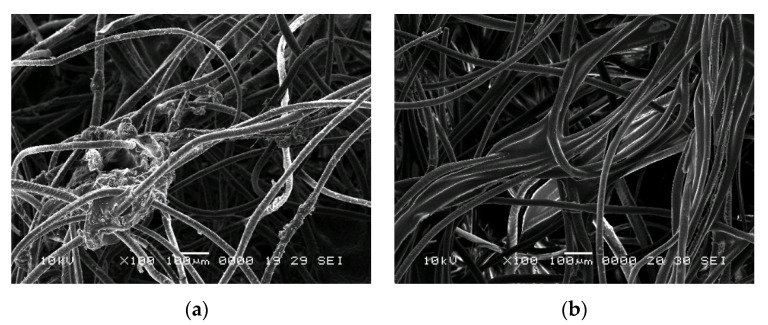
SEM images of wool-binder samples at ×100 magnification: (**a**) P3; (**b**) P13.

**Figure 13:**
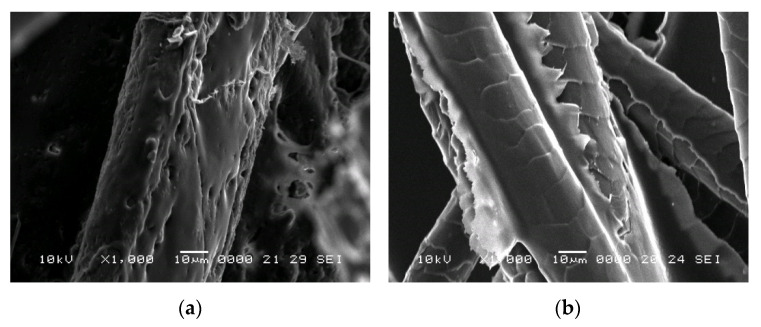
SEM images of wool-binder samples at ×1000 magnification: (**a**) P3; (**b**) P13.

In Figure 12 and Figure 13, SEM images of samples prepared with latex (P3) and resin (P13) are shown.

From the previous figures, the dispersion of the binder over the fibres can be seen, with possible agglomerations of the binder (case of sample P3). Analysing the samples at higher magnifications, binder exfoliation from the fibres and fibres partially covered by a binder can be seen (case of sample P13).

### 3.3. Chemical Characteristics

**Figure 14:**
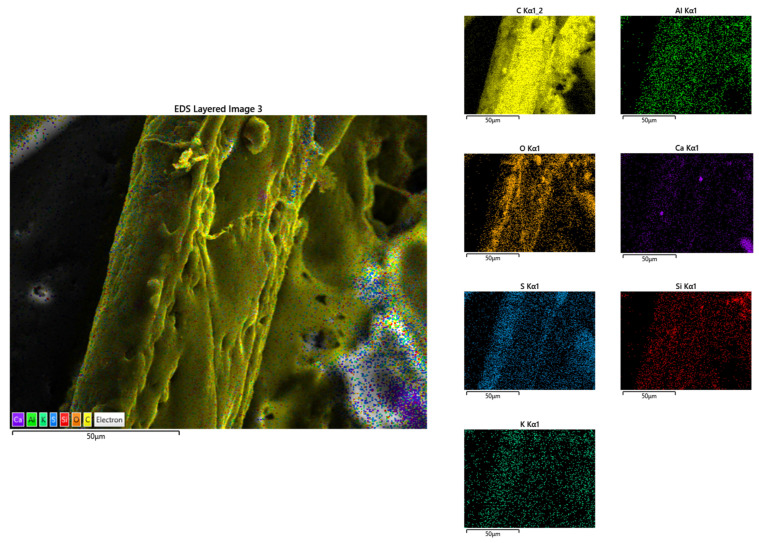
EDX elemental map distribution image of sample P3, magnification ×1000.

**Figure 15:**
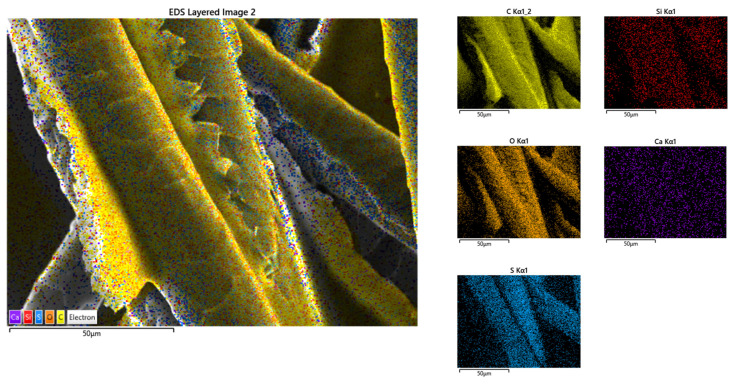
EDX elemental map distribution image of sample P13, magnification ×1000.

In Figure 14, the EDX elemental map distribution image of sample P3 is shown. On the basis of the results, the following component elements were identified: carbon, oxygen, sulphur, aluminium, calcium, silicon, and potassium. In Figure 15, EDX results for sample P13 are shown. For this sample, the elements carbon, oxygen, sulphur, silicon, and calcium were identified.

The homogeneous dispersion of the binder on the fibres can be seen in the previous figures. In both cases, the binder components were identified on the surface of the fibres, which confirms the previous statement. Based on this, the methods of making the samples (spraying binder over fibres for P3, mixing fibres with a binder for P13) are viable solutions for processing composites.

### 3.4. Water Absorption

**Figure 16:**
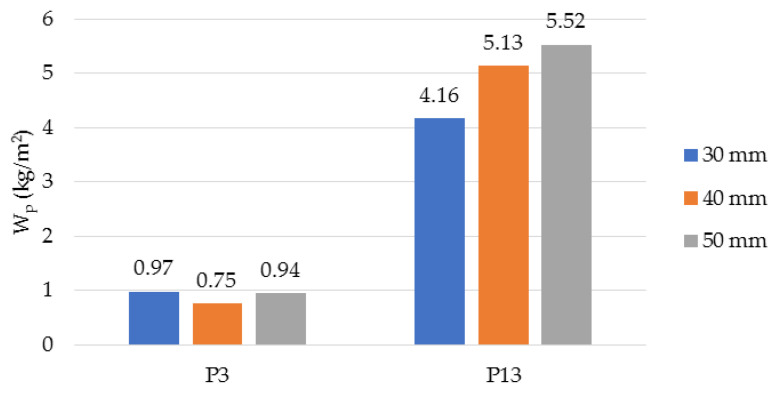
Short-term water absorption by partial immersion, W_p_, for samples P3 and P13.

In Figure 16, the result of the determination of short-term water absorption by partial immersion (W_p_) for the analysed samples, P3 and P13, is shown. For the sample made with latex (P3), the value of W_p_ is much lower than for the sample made with resin (P13). The influence of thickness on water absorption is more evident in the case of P13: the greater the thickness of the sample, the higher the water absorption value. For P3, the variation of water absorption with thickness is nonlinear, and the highest value was obtained for the minimum thickness.

### 3.5. Action of Microorganisms

**Figure 17:**
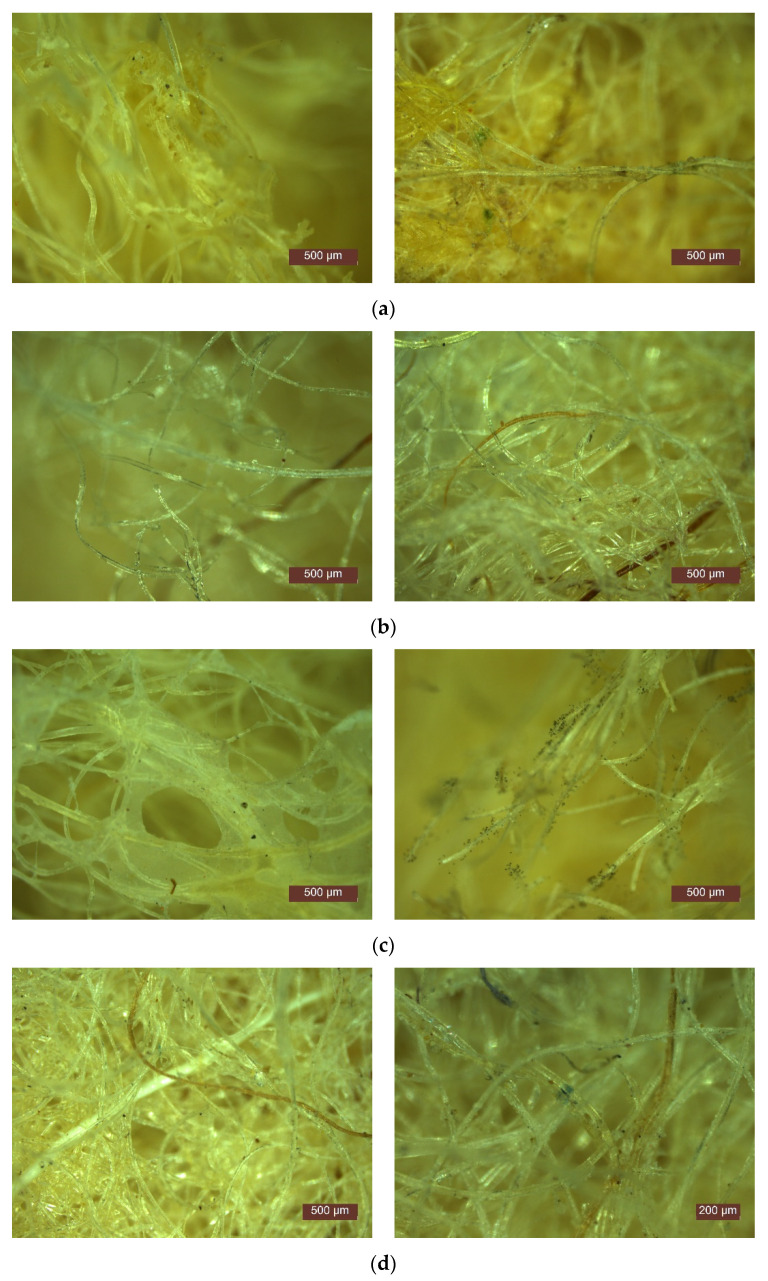
Growth of microorganisms identified on samples: (**a**) P3 after 4 weeks; (**b**) P13 after 4 weeks; (**c**) P3 after 6 weeks; (**d**) P13 after 6 weeks.

The results of the determination, i.e., the microscope images, are shown in Figure 17 for samples P3 and P13.

From the images presented, the presence of microorganisms on the samples analysed can be observed, but their identification is not the objective of this work. Regardless of the nature of the binder, the two types of wool samples exposed in the test environment show traces of microorganisms. Their increase is more pronounced as the exposure time increases.

The growth intensity of the microorganisms was assessed according to predetermined criteria. It was concluded that samples P3 and P13 fall into category 2 of microorganism growth intensity; therefore, the materials are not resistant to microorganism attack and contain nutrients for microorganism growth.

### 3.6. Water Vapour Transmission

In Table 3, the results of the water vapour transmission determination are presented. With respect to the μ-factor (water vapour diffusion resistance factor), both tested specimens show low values. Of the two types of specimens, the lowest value was obtained for the one prepared with latex (P3).

### 3.7. Hygroscopic Adsorption Characteristics

**Figure 18:**
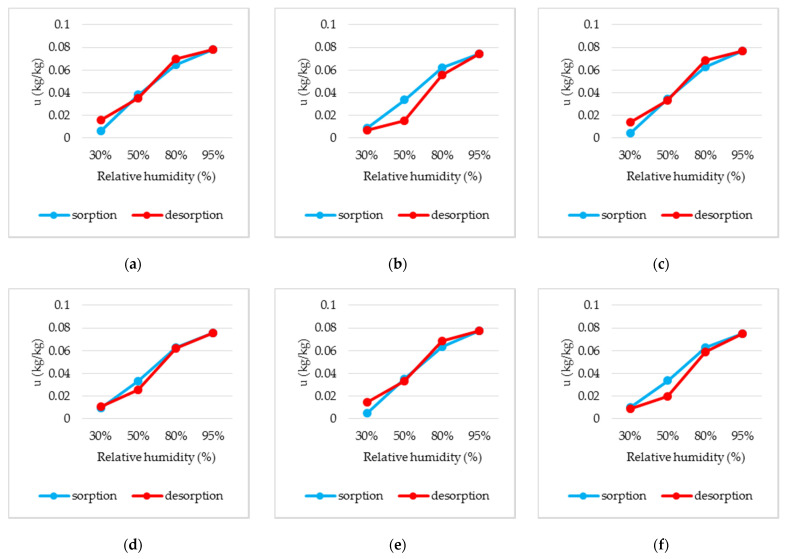
Sorption and desorption curves for: (**a**) P3-3 (wool–latex, thickness 3 cm); (**b**) P13-3 (wool–resin, thickness 3 cm); (**c**) P3-4 (wool–latex, thickness 4 cm); (**d**) P13-4 (wool–resin, thickness 4 cm); (**e**) P3-5 (wool–latex, thickness 5 cm); (**f**) P13-5 (wool–resin, thickness 5 cm).

In Table 4 and Figure 18, the results of hygroscopic adsorption determination for 3 cm (P3-3, P13-3), 4 cm (P3-4, P13-4) and 5 cm (P3-5, P13-5) thick samples are shown for the wool–resin (P13) and wool–latex (P3) composites. It is found that the values obtained for sample P13 (wool–resin) are defined by a hysteresis variation, while the sorption and desorption values measured for P3 (wool–latex) are closer, the difference between the two stages being small. For P3, in general, the sorption value does not exceed the desorption value, but for P13, sorption is higher than desorption.

The moisture content of sample P3, as a function of relative air humidity, varies between 0.0045 and 0.078 kg/kg, and for P13 this value is between 0.007 and 0.0755 kg/kg.

### 3.8. Sound Absorption

**Figure 19:**
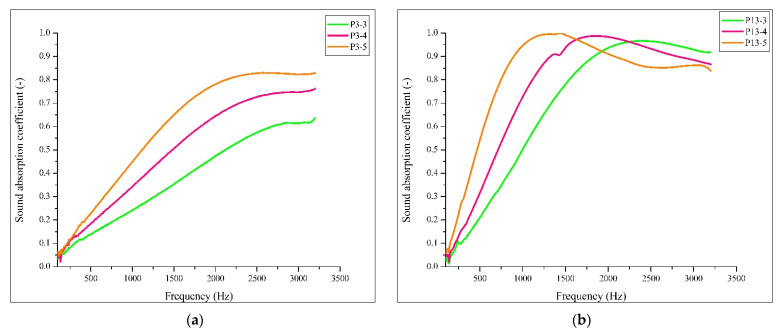
Sound absorption curves of samples: (**a**) P3 (wool–latex) with thicknesses of 3, 4 and 5 cm; (**b**) P13 (wool–resin) with thicknesses of 3, 4 and 5 cm.

In Figure 19 and Table 5, the results of the sound absorption determination are presented. The test was performed for samples P3 and P13, with thicknesses of 3 cm (P3-3, P13-3), 4 cm (P3-4, P13-4) and 5 cm (P3-5, P13-5).

Comparing Figure 19a,b, in terms of sound absorption, it can be seen that P13 (wool–resin) performs superior to P3 (wool–latex). Higher α_max_ values were measured for P13 in each thickness category. In the case of P3, the variation of the sound absorption coefficient is almost linear up to 2000 Hz, with maximum values being reached at 3200 Hz for the three specimen thicknesses. The peak sound absorption shifted to lower frequencies with increasing specimen thickness for both specimen types. Analysing the results of the 5-cm-thick samples, it can be seen that P13-5 shows high sound absorption over the whole frequency range analysed. In contrast, the P3-5 sample is more efficient at high frequencies.

## 4. Discussion

### 4.1. Thermal Conductivity

In general, high-density values imply high thermal conductivity values. Such results have been recorded for several natural fibres, such as bamboo [33], rice straw [34], hemp [35], or jute fibres [36]. However, for some materials, increasing density leads to low values of thermal conductivity. This phenomenon has been observed for hemp fibres [37], straw [38], reed [39], sheep wool [40], feathers [41], synthetic polymer fibres [42] and ceramic fibres [43]. This type of correlation between density and thermal conductivity can be explained in different ways. According to Pruteanu [38], reducing the volume of air in the mass of the material has the effect of reducing convective heat transfer, resulting in lower thermal conductivity values. Similar conclusions were formulated by Asdrubali et al. [39]. According to Ye et al. [40], the material’s structure or the test method could produce this type of correlation.

In the case of some natural fibre reinforced composites, it has been observed that by increasing the fibre dosage, the thermal conductivity value initially increases and then starts to decrease. If the optimum density point is exceeded, the tubular structure of the fibres becomes more deformed by compacting the composite. Thus, it was found that for flax fibres, increasing the sample density by using a higher number of fibres in the composite results in a decrease in thermal conductivity. The contribution of the fibres to the thermal insulating capacity is not manifested by their tubular structure but by the densification of the microfibrils that contribute to the thermal energy dissipation [44].

According to the study carried out on jute fibre-reinforced polymer composites, increasing the percentage of fibres in the composite leads to low thermal conductivity. Increasing the percentage of fibres implicitly increases the density of the composite, resulting in increased contacts between fibres, which explains the low values of thermal conductivity [45].

Kosiński et al. [46] studied the thermal insulation properties of hemp fibres and observed an inversely proportional variation between thermal conductivity and density. The authors came to the observation that any material can be characterised by an optimum density point for which the thermal conductivity is minimum.

The study by Stapulionienė et al. [47] on the influence of manufacturing technology and density on the thermal conductivity of natural fibres (flax and hemp) shows that the variation of thermal conductivity is governed by the optimum point of density. Before reaching the optimum density, the thermal conductivity is inversely proportional to the density, and after exceeding the optimum point, the variation is proportional or negligible.

Of the components of heat transfer in fibrous materials, radiation has the highest contribution, followed by conduction through air and conduction through fibres [48]. Conduction is directly proportional to density due to the high contact between fibres, but radiation varies inversely proportional to density due to the decrease in air volume in the material [43]. Since the decrease in the radiation component is greater than the increase in the conduction component, the total conductivity of the material decreases with increasing density [49].

The thermal conductivity values for wool panels obtained in this study range from 0.033 to 0.044 W/mK. Compared to these values, those identified in the literature are generally higher. Bosia et al. [50] studied a recycled wool-based panel with a thermal conductivity value of 0.040–0.054 W/mK. Ye et al. [40] obtained thermal conductivity coefficient values of wool panels of 0.034–0.067 W/mK. Patnaik et al. [17] obtained thermal conductivity values between 0.032 and 0.033 W/mK wool–polyester composites. Guna et al. [20] studied wool–polypropylene panels with λ values between 0.058 and 0.083 W/mK. Rubino et al. [19], for wool–chitosan samples, obtained λ values of 0.049–0.060 W/mK.

**Figure 20:**
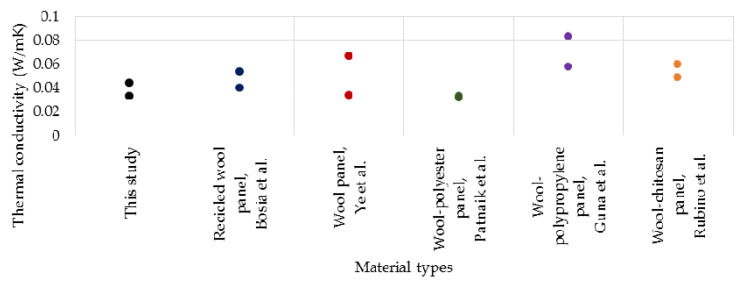
Thermal conductivity of samples; values of this study correspond to the minimum and maximum values recorded (data from refs. [17,19,20,40,50]).

In Figure 20, the thermal conductivity results of this study are presented, with reference to relevant materials found in the literature.

### 4.2. Water Vapour Transmission

The water vapour diffusion resistance factor (μ) is a parameter that shows the ability of a material to resist the transfer of water vapour through its mass. The lower this value is, the material is more permeable.

**Figure 21:**
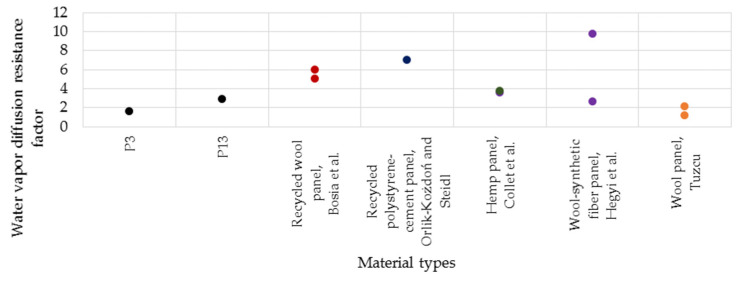
Water vapour diffusion resistance factor of samples (data from refs. [50,51,52,53,54]).

The μ-factor of the analysed samples is 1.6 for P3 (made with latex) and 2.9 for P13 (made with resin). These values are comparable or lower than those reported in the literature. Orlik-Kożdoń and Steidl [51] studied a porous thermal insulation material based on recycled polystyrene and cement. The studies showed that the water vapour diffusion resistance factor of the tested material was seven. Collet et al. [52] studied the properties of hemp-based composites and obtained μ-factor values of 3.6 and 3.7. Hegyi et al. [53] studied a sheep wool-based thermal insulation material in terms of water vapour behaviour. The authors recorded μ-factor values between 2.6 and 9.7. Tuzcu [54] measured μ-factor values between 1.20 and 2.12 for sheep wool. Bosia et al. [50] developed a recycled wool panel with a μ-factor of 5–6. In Figure 21, the comparative analysis of the μ-factor obtained in this study and the relevant results from the literature are reported.

### 4.3. Hygroscopic Adsorption Characteristics

The hygroscopic adsorption of wool fibres consists of two phenomena: moisture sorption followed by moisture desorption.

The results obtained in this study are in contradiction with those reported in the literature, where results were given for wool fibres without a binder. When the desorption curve is higher than the sorption curve (cases reported in the literature), the amount of water lost is higher than that adsorbed. When the sorption curve is above the desorption curve (the case obtained in this study), it indicates that there is an accumulation of moisture in the mass of the material. The fact that the fibres are coated with binder may influence the rate and degree of sorption and desorption, which could result in unusual hygroscopic adsorption characteristics.

Most of the surface of the fibres is covered with binder, so the adsorption phenomenon of the fibres is slightly hindered. Because of this, the difference between the minimum and maximum value of the moisture content of the samples is quite small, which can also lead to this inverse phenomenon of the curves’ variation.

**Figure 22:**
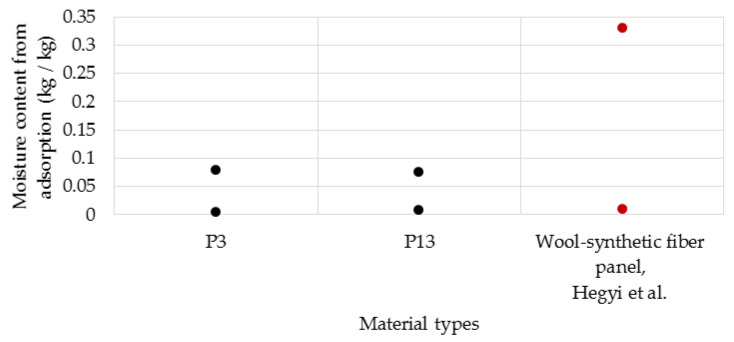
Moisture content from adsorption of samples (data from ref. [53]).

The hygroscopic adsorption of the materials analysed in this study ranges from 0.0045 to 0.078 kg/kg for P3 (made with latex) and 0.007 to 0.0755 kg/kg for P13 (made with resin). Hegyi et al. [53], in their study on thermo-sensitive synthetic fibre and wool fibre-based materials, reported values of adsorbed water between 0.01 and 0.33 kg/kg, depending on relative humidity. In Figure 22, the result obtained in this study in comparison with those reported by Hegyi et al. [53] is presented.

### 4.4. Sound Absorption

The sound absorption coefficient values of the panels made in this study, considering the maximum thickness of 50 mm of the specimen, are 0.83 for P3 (made with latex) and 0.99 for P13 (made with resin). Considering the values identified in the literature and the values measured in this study, it is observed that the latter ones are higher. Guna et al. [20] studied a material based on wool fibres and polypropylene fibres (30 mm thickness) with a maximum sound absorption coefficient of 0.86 between 1500 and 2500 Hz. Broda and Bączek [55] investigated the properties of wool samples (10.6 mm thickness), measuring a maximum sound absorption value of 0.95 at 3150 Hz.

**Figure 23:**
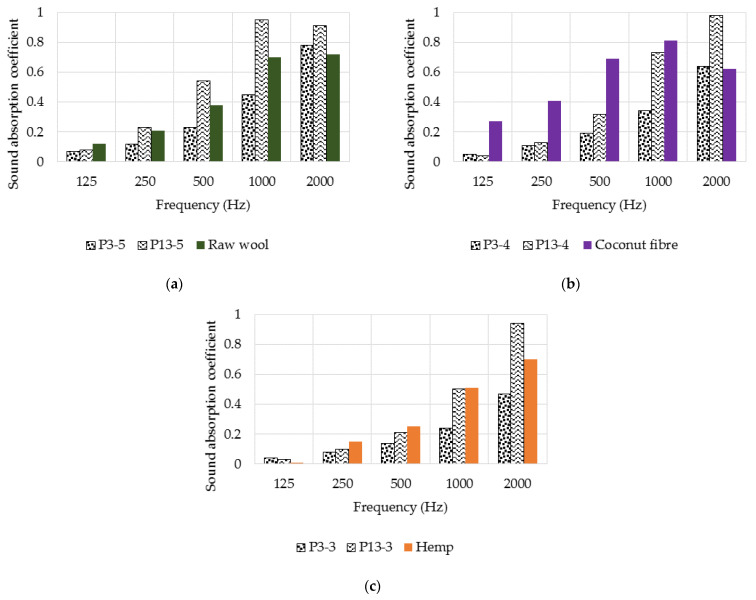
Sound absorption coefficients as a function of frequency for materials with different thicknesses: (**a**) 50 mm; (**b**) 40 mm; (**c**) 30 mm (data from refs. [56,57,58]).

With respect to the NRC value and sound absorption coefficients at standardised frequencies, the comparison between the materials studied in this experimental program and other fibrous materials is shown in Table 6 and Figure 23.

From the previously presented data, it can be seen that the sound absorption performance of the composites presented in this study is variable when it is compared with other natural fibrous materials. Comparing the samples with raw wool (the samples having 50 mm thickness), it can be observed that for frequencies between 250 and 1000 Hz, sample P13 has higher sound absorption, while the values of P3 are lower. Taking into consideration the case of coconut fibres (with samples having 40 mm thickness), it can be seen that the wool–binder composites have lower sound absorption in the range of 125–1000 Hz. Finally, from the relationship between the wool–binder samples and the hemp fibres (for 30 mm thick samples), it could be noted that the sound absorption values are similar between the frequencies of 12 and 1000 Hz. Overall, it can be noted that sample P13 in all three cases has higher acoustic absorption than raw wool, coconut fibres, and hemp at 2000 Hz by 19, 36 and 24%, respectively.

## 5. Conclusions

In this paper, different characteristics of sheep wool-based composites made with synthetic resin and natural rubber latex, respectively, were presented. In the first stage of the study, the thermal conductivity coefficient values of the composites were determined. The influence of some parameters (thickness, density, type of binder, percentage of binder) on the variation of the value of thermal conductivity and thermal resistance was investigated. In the second step, choosing two types of composites, the microscopic and chemical properties, water absorption, action of microorganisms, water vapour transmission, hygroscopic characteristics and acoustic absorption were determined.

Based on the results presented in this study, the following conclusions can be drawn:

- Increasing density has a different effect on the two types of binder: two minimum conductivity points were recorded for latex (at densities of 62.03 and 116.27 kg/m^3^), while one minimum point was measured for resin (at a density of 98.27 kg/m^3^);
- In general, resin leads to higher thermal conductivity values compared to latex;
- The increase in thermal conductivity is proportional to the increase in binder percentage;
- The minimum values of thermal conductivity were measured for the samples prepared with latex, having high density, with low binder percentage: 0.0324 W/mK (for sample P19 made by mixing the fibres with latex, in percentage of fibre/binder of 1/2, with density of 116 kg/m^3^) and 0.0331 W/mK (for sample P23 made by mixing fibres with latex, in percentage of fibre/binder of 1/2, with density of 138.74 kg/m^3^);
- Based on the criteria established in the national normative C107/2-2005 regarding the necessary values of thermal conductivity and thermal resistance (thermal conductivity less than 0.065 W/mK and thermal resistance greater than 0.50 m^2^K/W) [7] of a thermal insulating material, it is observed that all samples prepared and analysed in this study meet these criteria;
- Microscopic and chemical analysis confirmed the dispersion of the binder on the fibres;
- Water absorption is higher in the resin sample;
- The materials tested are susceptible to attack by microorganisms;
- The water vapour diffusion resistance factor is higher for the wool–resin sample;
- The wool–resin sample shows high sound absorption over the whole frequency range analysed, while the wool–latex sample is more efficient at high frequencies.

However, this study has a few limitations, but they will be covered in the next research program, which will aim at finding solutions to the limitations met at this stage. Firstly, the investigation methodologies will cover tests of compositional analysis techniques, such as X-ray photoelectron spectroscopy, Fourier-transform infrared spectroscopy and thermogravimetric analysis, but it will also focus on determining other physical properties, such as the sound and impact insulation, fire resistance or in situ hygrothermal monitoring. Secondly, the question of automating the production process of the samples to reduce the preparation time will be investigated. In order to propose a viable alternative to the common insulation materials, this aspect should be covered. Thirdly, the economic aspect will be taken into consideration, which is decisive when it comes to creating a building material. Overall, the experimental part will be expanded, but the practical and economic sides of these materials will be assessed as well.

Since this experimental program is the result of the first stage of researching the presented composites, the following stages will be centred around developing them in order to meet the three criteria of sustainability.

## Figures and Tables

**Table 1:** The characteristics of the resin and latex, according to the manufacturer.

Property	Latex	Resin
pH	9.8–10.8	7.5–8
Water solubility	Yes	Yes
Density	0.976 g/cm^3^	1.035–1.045 g/cm^3^
Solids content	60.5%	-
Ammonia content	0.6%	-
Content of non-volatile substances	-	30 ± 1%
Content of volatile organic compounds	-	70 g/L

**Table 2:** Recipes of materials for wool-binder samples.

Recipe	Preparation Method	Wool Fibre Density in the Composite (g/cm^3^)	Binder Type	Fibre:Binder Weight Percentage
P1	Spraying	0.030	Resin	1:2
P2				1:4
P3			Latex	1:2
P4				1:4
P5		0.050	Resin	1:2
P6				1:4
P7			Latex	1:2
P8				1:4
P9		0.070	Resin	1:2
P10				1:4
P11			Latex	1:2
P12				1:4
P13	Mixing	0.078	Resin	1:2
P14				1:4
P15			Latex	1:2
P16				1:4
P17		0.096	Resin	1:2
P18				1:4
P19			Latex	1:2
P20				1:4
P21		0.114	Resin	1:2
P22				1:4
P23			Latex	1:2
P24				1:4

**Table 3:** Water vapour transmission factors: g—water vapour transmission rate; W—water vapour permeance; Z—water vapour resistance; δ—water vapour permeability; μ—water vapour diffusion resistance factor; s_d_—water vapour diffusion equivalent air layer thickness.

Sample	g (mg/m^2^·h)	W (mg/m^2^·h·Pa)	Z (m^2^·h·Pa/mg)	δ (mg/m·h·Pa)	μ	s_d_ (m)
P3	1.11·10^4^	9.16	0.1092	0.45	1.6	7.75·10^−2^
P13	6.00·10^3^	4.96	0.2015	0.24	2.9	1.43·10^−1^

**Table 4:** Moisture content values of samples for relative humidity of 30, 50, 80 and 95%.

Sample	Moisture Content, u (kg/kg)							
	Sorption				Desorption			
	30%	50%	80%	95%	95%	80%	50%	30%
P3-3	0.0065	0.0380	0.0645	0.0780	0.0780	0.0695	0.0350	0.0160
P3-4	0.0045	0.0345	0.0630	0.0770	0.0770	0.0685	0.0330	0.0140
P3-5	0.0050	0.0350	0.0635	0.0775	0.0775	0.0685	0.0335	0.0145
P13-3	0.0090	0.0335	0.0620	0.0745	0.0745	0.0555	0.0150	0.0070
P13-4	0.0095	0.0330	0.0625	0.0755	0.0755	0.0620	0.0255	0.0105
P13-5	0.0100	0.0335	0.0630	0.0750	0.0750	0.0590	0.0200	0.0090

**Table 5:** Maximum sound absorption coefficient (α_max_), frequency corresponding to the maximum sound absorption, and the noise reduction coefficient (NRC) for the tested samples.

Parameter	P3-3	P3-4	P3-5	P13-3	P13-4	P13-5
α_max_	0.636	0.762	0.830	0.966	0.988	0.999
Frequency of α_max_ (Hz)	3200	3200	3200	2364	1864	1436
NRC *	0.234	0.321	0.394	0.437	0.540	0.657

* NRC was calculated according to ASTM Standard C423 [32].

**Table 6:** Sound absorption coefficients as a function of frequency, and NRC values for different materials.

Material	Thickness (mm)	Frequency (Hz)					NRC	Ref.
		125	250	500	1000	2000		
P3-5	50	0.07	0.12	0.23	0.45	0.78	0.39	This study
P13-5	50	0.08	0.23	0.54	0.95	0.91	0.66	
P3-4	40	0.05	0.11	0.19	0.34	0.64	0.32	
P13-4	40	0.04	0.13	0.32	0.73	0.98	0.54	
P3-3	30	0.04	0.08	0.14	0.24	0.47	0.23	
P13-3	30	0.03	0.10	0.21	0.50	0.94	0.44	
Raw wool	50	0.12	0.21	0.38	0.70	0.72	0.50	[56]
Coconut fibre	40	0.27	0.41	0.69	0.81	0.62	0.63	[57]
Hemp	30	0.01	0.15	0.25	0.51	0.70	0.40	[58]

## Data Availability

The data presented in this study are available on request from the corresponding authors.
